# Cases of Typhus Fever, in Which the Affusion of Cold Water Has Been Applied in the London House of Recovery

**Published:** 1803-03-01

**Authors:** W. P. Dimsdale


### Cases of TYPHUS FEVER, in which the AFFUSION of COLD WATER has been applied in the LONDON HOUSE of RECOVERY. By W. P. DIMSDALE, M. D.

CASE I. JAMES JOHNSON, aged eight years, caught the infection from his parents, who died of fever. He was removed on the 19th May, 1802, into the House of Recovery. On the 23d of May (the twelfth day of the disease), the symptoms were as follow:—pulse extremely frequent; tongue covered with a dark fur, and very dry; skin dry. A thermometer placed under the tongue arose to 104°: constant and violent delirium. The usual medical treatment not being attended with success, recourse was had to the affusion of cold water. He was taken out of bed, stripped, and a pitcher of cold water was poured suddenly over him: After being wiped, he was replaced in bed. He slept an hour; the skin felt more relaxed; no perspiration however followed. May 24th, pulse 120; skin dry; heat 100°; delirium continues; no sleep in the night. The affusion was repeated with a pail of cold water. He again slept quietly, was evidently more collected when he awoke; and soon afterwards a profuse perspiration came on, which continued through the night. On May 27th (the fourth day after the cold affusion had been first used), he was entirely free from fever.

CASE II. Thomas Knight, aged twelve years, was admitted June 16th, on the fifth or sixth day of *typhus*. In the the afternoon, pulse 116; skin dry, with numerous *petechiæ*; heat 104°; eyes suffused; violent pain of the head. The cold affusion, with a pail of water, was directed. The pain of the head subsided, he slept quietly, and copious perspiration followed. From this time the symptoms were favourable. On the 22d he was free from disease, on the fourth day after he was removed into the house.

CASE III. John Harrogan, aged twenty-six years, came into the house on July 8th, the fifth day of the disease: pulse 120; tongue furred and dry; skin hot and partially moist; delirious at intervals; pain of the head and back. July 9th, violent delirium came on in the night, two nurses were unable to keep him in bed. The matron of the house sent for me at five o'clock this morning; he was then very outrageous; pulse 136; skin hot and parched. He was placed by force under the shower-bath, and two pails of cold water were poured instantly over him. The transition from a state of extreme fury to perfect calmness, was truly surprising. Without an effort of resistance on his part, he was replaced in bed: profuse perspiration succeeded. In three days he had no symptom of fever remaining.

CASE IV. Alfred Sweeting, aged four years, was removed into the house 13th July: he caught the infection from his mother, who died in a small and dirty apartment. On July 15th, fourth day of the disease, pulse very frequent, skin dry, heat 102°; tongue slightly furred, countenance expressive of much uneasiness. The shower bath was used: he appeared immediately to be much relieved; general moisture of the skin followed. On the 16th he was free from fever. This patient took only the saline mixture, and afterwards small doses of the diluted nitrous acid.

CASE V. Henry Hancock, aged twenty-eight years, was on the 10th of August, the fifth day of *typhus*, removed into the house. Pulse 120; tongue furred, slightly moist; skin very dry; heat 105°; severe pain of the head. The shower bath was directed. The pain of the head was removed instantly: Perspiration succeeded. The symptoms continued favourable to the 14th, when he had no complaint remaining except weakness.

CASE. VI. George Johnson, aged fifteen years, came in on the 13th of Angust. On the 14th, (fifth day of *typhus*), pulse 124, heat 98°; slight partial moisture of the skin; skin; the tongue furred, and much general uneasiness. August 15th, he has been very delirious in the night, and extremely restless: complains of violent pain of the head: pulse very frequent, tongue furred, rather dry; skin dry, numerous *petechiæ* over the body; heat 103°. The shower bath was immediately used. The pain of the head was instantly removed, but no general perspiration followed. In the evening the head-ach and the other febrile symptoms returned with nearly the same severity as before. The cold affusion wss again used, and he felt immediate relief. Copious perspiration very soon succeeded, which continued through the night. He was free from complaint on the 17th, the third day after the first use of the cold affusion.

Case VII. John Beard, a boy aged eleven years, was admitted on the 21st of August, in the third day of fever, with the usual symptoms: pulse frequent; much thirst; pain of the head and back; the skin rather moist. 22d, skin dry, heat 103°, pulse 116, tongue furred; pain of the head continues. The cold affusion was directed immediately; and applied again in the evening. He passed the night easily; the skin was partially moist: he had some refreshing sleep. August 23d, the skin is now dry; heat 104°; complains as before of much pain and general uneasiness. He again used the shower both. In the evening, the skin being dry, and the heat 102°, it was repeated: profuse perspiration came on in the night. 24th, skin very moist; heat 98°; pulse 100; tongue slightly furred; says he feels much better. In the evening, during a short absence of the nurse, feeling a slight return of heat and uneasiness, he poured a pitcher of cold water which was in the room over himself into the bed. The nurse returning immediately, she removed him to a dry bed; he slept quietly through the night, the skin moist, and awoke in the morning quite free from fever. The only medicines, ordered in this case, were the saline mixture, and small doses of colombo.

Case VIII. Abraham Johnson, aged twenty years, was admitted on the 6th of September, with the usual symptoms, in the fifth day of fever. Sept. 7th, skin dry; heat 100°. The shower bath was used, which produced considerable relief. On the 9th the heat was again 100°; the skin dry. The cold affusion was repeated. He was free from fever on the 12th.

Case IX.

CASE IX. Mary Johnson, aged eleven years; removed into the house August 13th, in a late period of fever. She relapsed August 22. On the 23d, pulse 132: tongue covered with a dark fur, rather dry; skin dry; heat 103°; pain of the head and back. Copious perspiration succeeded the cold affusion, and in two days she was entirely free from fever.

CASE X. Robert Holmes, aged twenty-six years; admitted September 7th, with fever of uncertain date. Pulse 100, tongue slightly furred, pain all over the body. In the evening the skin became very dry; heat 100°. A slight delirium with which he was affected, subsided immediately on the use of the shower bath. He was free from fever on the 10th, but extremely feeble. By the use of a nourishing diet, with small doses of the bark and wine, he gradually regained his former strength.

CASE XI. John Dutchfield, aged twenty-one years; admitted on the 25th of September, with the usual symptoms of fever. On the 28th (ninth day of the disease), skin dry, heat 100°; used the shower bath; the heat diminished, the skin became moist. On the 2d of October he was free from fever.

CASE XII. Mary Simmons, aged forty-two years, was admitted November 18th into the house, with the usual symptoms of fever; the date uncertain. On the 20th, pain of the head exceedingly violent, skin dry, heat 99½°. The head-ach ceased immediately after the cold affusion, the skin became rather moist. On the 23d, the heat again rose to 99½; the skin dry; copious perspiration followed a repetition of the affusion. She was free from fever on the 25th.

### OBSERVATIONS.

It appears unnecessary to relate the other cases in which the cold affusion has been used. In all, the good effects of it have been strikingly manifest, and in no instance has the disease terminated fatally after the use of this remedy. In the early stages of *typhus*, the affusion, with very little assistance from medicine, appears to cut short the progress of the disease. In the more advanced periods, when the strength of the patient is sufficient to admit the application of this remedy, it moderates the violence of the symptoms, and contributes materially towards a favourable termination.

termination. When the strength is greatly exhausted, it may probably be wholly inadmissible. The patients almost invariably expressed great satisfaction, after the agitation immediately following the affusion had subsided. The violent pain of the head, so distressing in fever, is almost constantly and immediately removed, and generally, quiet sleep succeeds, with moisture of the skin.

Case the 7th furnishes a strong illustration of these remarks. The boy always, after the first affusion, went to the bath with perfect readiness, and even solicited its repetition. The almost immediate discovery of the affusion which he had himself practised, prevented any injurious consequences; and it is evident from the report of the following day, that the slight exacerbation of fever* which came on in the evening, was completely moved by this application. The feelings of the patient in this instance immediately prompted him to have recourse to the remedy, from which he had before experienced so much relief.

Spring water has been used hitherto without any addition. A shower-bath is placed in the House of Recovery, for the purpose of applying the remedy. It is obvious that the affusion is by this means rendered more complete than by any other mode of application; it is also neater, and more commodious. Ablution of the body, by spunging with cold or tepid water and vinegar, has been frequently employed with advantage: it is however less effectual than the affusion.

I shall feel peculiar gratification if this short account, by confirming the facts stated in the elegant and truly valuable publication of Dr. Currie, should tend to accelerate the general introduction of a remedy so important in the treatment of fever; being fully convinced from the uniform success which has attended the practice, that it may be used with perfect safety in this disease, "when (to use Dr. Currie's words,) there is no sense of chilliness present, when the heat of the surface is steadily above what is natural, and when there is no general or profuse perspiration."

*5th Jan.* 1803.

---

* The reader, who is desirous of information as to the use of the *cold affusion* (or of the *tepid bath*) in cases of *scarlet fever*, is referred to Dr. Currie's Medical Reports, p. 60, 61, and 62; and to some other parts of that excellent work.

